# The AAA+ proteins Pontin and Reptin enter adult age: from understanding their basic biology to the identification of selective inhibitors

**DOI:** 10.3389/fmolb.2015.00017

**Published:** 2015-05-05

**Authors:** Pedro M. Matias, Sung Hee Baek, Tiago M. Bandeiras, Anindya Dutta, Walid A. Houry, Oscar Llorca, Jean Rosenbaum

**Affiliations:** ^1^Instituto de Tecnologia Química e Biológica António Xavier, Universidade Nova de LisboaOeiras, Portugal; ^2^Instituto de Biologia Experimental e TecnológicaOeiras, Portugal; ^3^Creative Research Initiative Center for Chromatin Dynamics, School of Biological Sciences, Seoul National UniversitySeoul, South Korea; ^4^Department of Biochemistry and Molecular Genetics, University of VirginiaCharlottesville, VA, USA; ^5^Department of Biochemistry, University of TorontoToronto, ON, Canada; ^6^Centro de Investigaciones Biológicas, Consejo Superior de Investigaciones Científicas (Spanish National Research Council, CSIC)Madrid, Spain; ^7^INSERM, U1053Bordeaux, France; ^8^Groupe de Recherches pour l'Etude du Foie, Université de BordeauxBordeaux, France

**Keywords:** TIP48, TIP49, RUVBL1, RUVBL2, Rvb1, Rvb2, chaperone, cilium

## Abstract

Pontin and Reptin are related partner proteins belonging to the AAA+ (ATPases Associated with various cellular Activities) family. They are implicated in multiple and seemingly unrelated processes encompassing the regulation of gene transcription, the remodeling of chromatin, DNA damage sensing and repair, and the assembly of protein and ribonucleoprotein complexes, among others. The 2nd International Workshop on Pontin and Reptin took place at the Instituto de Tecnologia Química e Biológica António Xavier in Oeiras, Portugal on October 10–12, 2014, and reported significant new advances on the mechanisms of action of these two AAA+ ATPases. The major points under discussion were related to the mechanisms through which these proteins regulate gene transcription, their roles as co-chaperones, and their involvement in pathophysiology, especially in cancer and ciliary biology and disease. Finally, they may become anticancer drug targets since small chemical inhibitors were shown to produce anti-tumor effects in animal models.

## Introduction

**Figure 1:**
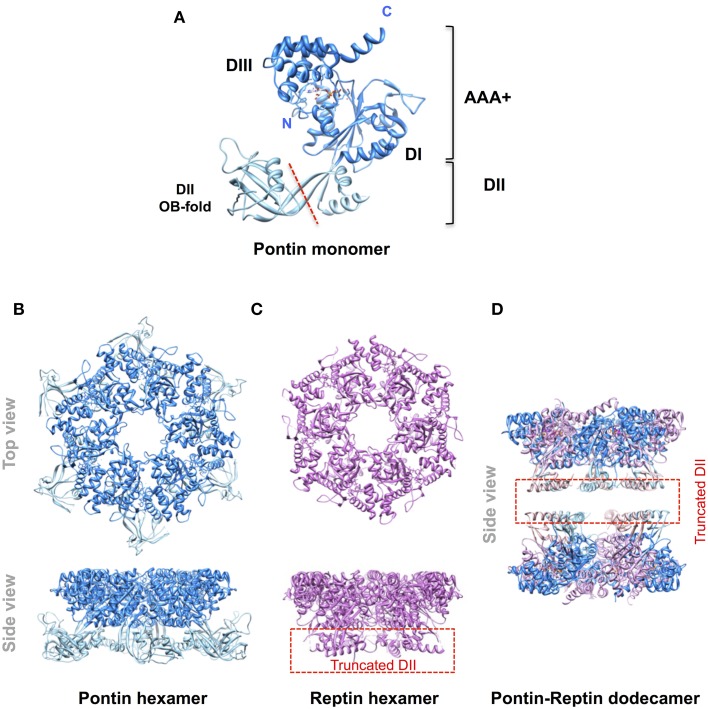
**Molecular organization of Pontin and Reptin**. Ribbon diagrams of **(A)** the Pontin monomer (PDB 2C9O, Matias et al., 2006) showing its domain structure; **(B)** the Pontin hexamer (*ibid*); **(C)**, a Reptin hexamer (PDB 3UK6, Petukhov et al., 2012); **(D)** a Pontin-Reptin dodecamer (PDB 2XSZ, Gorynia et al., 2011). In **(C,D)** the domain II region to the left of the red dashed line in **(A)** was truncated to facilitate crystallization. Molecular structures were drawn using the UCSF Chimera software (Pettersen et al., 2004).

Pontin and Reptin are related partner proteins belonging to the AAA+ (ATPases Associated with various cellular Activities) family. They came to attention in the late 1990s and it became rapidly clear that they were implicated in multiple and seemingly unrelated processes encompassing the regulation of gene transcription, the remodeling of chromatin, DNA damage sensing and repair, and the assembly of protein and ribonucleoprotein complexes among others (Gallant, 2007; Huber et al., 2008; Jha and Dutta, 2009; Cheung et al., 2010; Nano and Houry, 2013). A second wave of interest arose when they were found overexpressed in many cancers and shown to play roles in tumor biology (Grigoletto et al., 2011). Pontin and Reptin share some homology with the bacterial RuvB helicase (hence their names RUVBL1/2) but possess an extra domain II (Figure 1). Similar to RuvB they have an ATPase activity but their helicase activity remains questioned. Both proteins can form oligomers like homo-hexamers, hetero-hexamers, or hetero-dodecamers (Figure 1). They were discussed during a First International Workshop held in Bordeaux, France, in 2012. It was a great success as it allowed scientists from various fields to meet for the first time, to discuss in depth their favorite proteins, and to share expertise and reagents. In particular, this Workshop led to a consensus in limiting the number of names used for these proteins to Pontin/RUVBL1 and Reptin/RUVBL2 in humans (or RVB1/Rvb1 and RVB2/Rvb2 in yeast) (Rosenbaum et al., 2013). However, many open questions remained, justifying a Second Workshop that was held in Oeiras, Portugal, in October 2014, of which selected parts form the basis of this Perspective article.

## Mechanisms of transcriptional regulation

**Figure 2:**
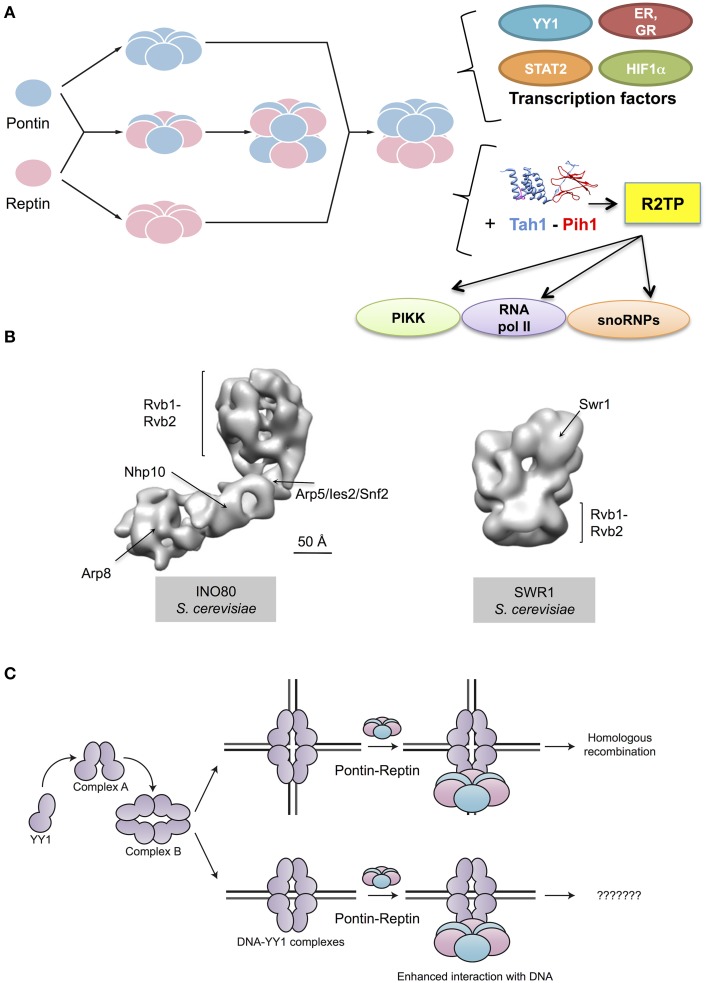
**Cellular functions of Pontin and Reptin. (A)** Pontin and Reptin in a yet unclear supramolecular organization interact and affect the transcriptional activity of several transcription factors. They directly interact with YY1 and increase its affinity for DNA, in a sequence-independent manner. Both proteins interact with STAT2 and with nuclear receptors (estrogen receptor, ER, and glucocorticoid receptor, GR) and act as agonists for transcription. Both also interact with HIF1α, and when methylated Reptin acts as a repressor but Pontin as an agonist of its transcriptional activity. They associate with Tah1 and Pih1 to form the R2TP complex that helps assemble snoRNPs, phosphatidylinositol-3-kinase-like kinases (PIKK) proteins and RNA polymerase II. Ribbon diagrams of Tah1 and Pih1 are based on PDB 4CGU (Pal et al., 2014). **(B)** Both proteins are also important for other complexes involved in the remodeling of chromatin: they help assemble the INO80 complex and are integral components of this complex. An intermediate resolution structure of INO80 has been recently determined using electron microscopy (EMD-2385 and 2386, Tosi et al., 2013). Within this structure of INO80, several subunits have been localized, including Rvb1 and Rvb2. Pontin and Reptin are also part of the SWR1 complex. An intermediate resolution structure of this complex obtained by electron microscopy revealed a ring formed by Rvb1 and Rvb2 functioning as scaffold for other subunits in the complex (EMD-5626, Nguyen et al., 2013). **(C)** Pontin and Reptin interact with oligomeric forms of YY1 transcription factor, which are labeled as complex A and complex B (Lopez-Perrote et al., 2014). The Pontin-Reptin-YY1 complex shows an enhanced affinity for DNA *in vitro*, but the relevance of this in the cell is unknown. The Pontin-Reptin-YY1 complex appears to participate in DNA repair by Homologous recombination (Lopez-Perrote et al., 2014).

Pontin and Reptin have long been known to be involved in the regulation of gene transcription. However, they are not themselves transcription factors. How they function besides their roles in chromatin remodeling remains to be clarified. This meeting brought new information on their interactions with bona fide transcription factors (Figure 2A).

David Levy (New York, U.S.A.) reported that Pontin and Reptin are required for the induction of interferon stimulated genes in response to interferon alpha, but not for the induction of genes by tumor necrosis factor alpha or interferon gamma (Gnatovskiy et al., 2013). Interferon stimulated genes (ISG) induction by interferon alpha is normally dependent on a nuclear ISGF3 transcription factor complex that is composed of tyrosine phosphorylated STAT1 and STAT2 together with the DNA binding subunit IRF9, which is recruited to the promoters of ISGs. Pontin and Reptin were found to interact with the transcriptional activating domain of STAT2, but were however not necessary for the recruitment of ISGF3 to the ISG promoter. Strikingly, it also appears that their ATPase activity is not required for ISG transcription.

Otmar Huber (Jena, Germany) reported on the role of Pontin and Reptin in the regulation of nuclear hormone receptors. He provided evidence that both proteins can bind to estrogen and glucocorticoid receptors. Here, as in the case of ISGs, Pontin and Reptin appear to have concordant effects on the nuclear hormone receptors, which is in contrast with the previously described antagonistic regulation of β-catenin/TCF transcriptional activity by Pontin and Reptin (Bauer et al., 2000; Rottbauer et al., 2002).

Sung Hee Baek's group (Seoul, South Korea) has worked on the role of Pontin and Reptin in the transcriptional regulation and chromatin remodeling in the context of cancer and stem cells. She reported that methylation of Pontin and Reptin affects their transcriptional activities in hypoxia signaling. In hypoxic conditions, Reptin is methylated by G9a, whereas Pontin is methylated by G9a and GLP (Lee et al., 2010, 2011). Reptin methylation participates in negative regulation of hypoxia target genes, but Pontin methylation hyperactivates a subset of hypoxia target genes. Genome-wide analyses showed that Pontin and Reptin do not share many common target genes in hypoxia signaling pathways. These studies support the model whereby Pontin and Reptin are crucial players in transcriptional activation and repression, respectively.

How do Pontin and Reptin, which are supposedly part of the same complexes, achieve specificity in transcriptional regulation requires further studies. It may be that for their transcriptional functions they do not function as hetero-oligomers, a hypothesis supported by some data using ChIP/re-ChIP showing that they are not found bound together to the same DNA sequences (Rowe et al., 2008).

## Chaperone activity

Pontin and Reptin participate to the R2TP (Rvb1-Rvb2-Tah1-Pih) complex (Kakihara and Houry, 2012). This complex is implicated in the assembly and stabilization of several macromolecular complexes, including small nucleolar ribonucleoproteins and phosphatidylinositol-3-kinase-like kinases. R2TP comprises several proteins that bind to Pontin and Reptin and it is thought to function as a co-chaperone of the HSP90 chaperone system. The structural and molecular biology of this complex and its interaction with HSP90 are however poorly characterized.

Mohinder Pal (Brighton, United Kingdom) described his efforts and those of the group of Laurence Pearl and Chrisostomos Prodromou to reveal the structural basis of the assembly of the human and yeast R2TP complex. He showed crystal structures of several sub-complexes, which, combined with biochemical and biophysical experiments allowed him to define the structural basis by which R2TP connects HSP90 to several substrates (Pal et al., 2014).

Walid A. Houry (Toronto, Canada) discussed the role of R2TP in the assembly of box C/D snoRNPs, which consist of a snoRNA and the four protein components Nop1, Nop56, Nop58, and Snu13. He described an unexpected finding in which the activity of the R2TP complex was required for Nop58 protein stability and is controlled by the dynamic subcellular redistribution of the R2TP complex in response to growth conditions and nutrient availability. In growing cells, the R2TP complex was found to localize to the nucleus and to interact with box C/D snoRNPs. This interaction was significantly reduced in poorly growing cells where R2TP predominantly relocalizes to the cytoplasm. The data presented showed that the R2TP complex exerts a novel regulation on box C/D snoRNP biogenesis that affects their assembly and consequently pre-rRNA maturation in response to different growth conditions (Kakihara et al., 2014).

Edouard Bertrand (Montpellier, France) also provided biochemical evidence for a snoRNP assembly pathway in mammalian cells that involves an RNA-free complex made of snoRNP core proteins, adaptor proteins, and Pontin/Reptin (Bizarro et al., 2014; Rothe et al., 2014). He proposed that the role of the R2TP complex is to load Pontin/Reptin onto this complex. Bérengère Pradet-Balade from the same group has recently characterized the *Drosophila* SPAG gene, which is homologous to mammalian RPAP3 and yeast Tah1 that are present in the R2TP complex (Benbahouche et al., 2014), and showed that RPAP3 in *Drosophila*, is necessary for embryonic development.

Finally, Pontin and Reptin are also involved in the assembly or stabilization of other protein complexes such as INO80 and SWR1 (Figure 2B). Anindya Dutta's group (Charlottesville, U.S.A.) previously reported that RVB proteins were required for Arp5 to be incorporated into the yeast Ino80 complex (Jonsson et al., 2004). The recent structure of the Ino80 complex presented by Karl-Peter Hopfner (Munich, Germany) provides support for this model by showing that Arp5-Ies6-RVB form a module that appears to cross-link to the Ino80 molecule only through RVB proteins (Figure 2B) (Tosi et al., 2013). Pontin and Reptin also interact by other means with INO80 since Oscar Llorca (Madrid, Spain) showed during this meeting that Pontin and Reptin interact with Yin Yang-1 (YY1), a transcription factor that in humans also forms part of the INO80 complex (Lopez-Perrote et al., 2014). *In vitro* experiments showed a direct interaction between YY1 and Pontin-Reptin. They showed that Pontin-Reptin complexes could target YY1 to DNA independently of the YY1 consensus sequence and increase its affinity for DNA (Figure 2C). Finally, Anastas Gospodinov (Sofia, Bulgaria) reported a new role for the mammalian INO80 complex in unchallenged replication and under conditions of replication stress where it is required to protect stalled forks and allow their subsequent restart (Vassileva et al., 2014). Whether this activity requires Pontin-Reptin is not known.

As shown by Dutta, the TIP60 lysine acetyltransferase complex is another chromatin remodeling factor that is dependent on the presence of Pontin-Reptin. In this case, however, these proteins appear to be individually important for stabilizing TIP60 and preventing a non-productive (perhaps inhibitory) interaction between p400, another component of the TIP60 complex and the catalytic TIP60 subunit (Jha et al., 2013).

It remains to be understood whether Pontin and Reptin help assemble INO80 and Tip60 in an R2TP-dependent or independent manner.

## Enzymatic activity

The nature and the mechanisms of the enzymatic activities of Pontin and Reptin are still very much questioned. This meeting brought some new information, which makes use of modeling and biophysical methods.

Based on the structural analysis of the single ancient Pontin/Reptin ortholog found in the archaeon *Methanopyrus kandleri*, Arina Afanasyeva (St. Petersburg, Russia) presented a model for the pre-transition state assembly of all components required for ATP hydrolysis to occur in the Pontin/Reptin heterohexamers (Afanasyeva et al., 2014). She analyzed effects of double-stranded DNA, docked within the central channel of the hexameric Pontin/Reptin rings, on the appearance of lytic water molecules in the close proximity of the ATP γ-phosphate. Her calculations suggest that the presence of DNA may significantly increase the probability of ATP hydrolysis via promotion of the appropriate hydrogen bonds formation between the lytic water molecules and the negatively charged residues located within the protein's catalytic pockets.

Rémy Bailly (Bordeaux, France) described the use of molecular dynamics simulations to study protein-protein interactions in Pontin-Reptin complexes. These simulations were based on preliminary studies led by virtual screening, which allowed the identification of small-molecule inhibitors of Pontin (Elkaim et al., 2012, 2014). All-atom molecular dynamics of the monomers revealed conformational changes dependent on the ligands ADP and ATP. These simulations highlighted the mobility of domain II, which induces a compact non-native conformation in the presence of ADP or an extended one with ATP. To study larger multimeric complexes, a mesoscopic approach called coarse-grain was used. The significant reduction in computational time in this model allowed new timescales to be reached (beyond the microsecond). The coarse-grain method was used to study dodecameric structures of Pontin and Reptin and suggested the formation of one side channel in the presence of ATP, whereas no channel appeared with ADP. In the context of a helicase activity, this channel would be large enough to allow the passage for a single strand DNA (26 Å) while the other strand goes through the central channel.

## Pathophysiology and therapy

This meeting has clearly demonstrated that Pontin and Reptin are no longer only exciting and sophisticated objects for fundamental studies, but are now implicated as regulators of essential processes in development and physiology, in the response to pathogens, and that their deregulation contributes to the pathogenesis of several diseases including cancer. Following pioneering academic attempts at designing inhibitors (Elkaim et al., 2012), this has led to the interest of big pharmaceutical companies, with highly active and selective inhibitors now nearing clinical trials.

Sara Cherry's laboratory (Philadelphia, U.S.A.) is interested in identifying antiviral genes that affect infection by RNA viruses transmitted to vertebrates by insect vectors. They identified dPontin (*Drosophila* Pontin) in a genome-wide RNAi screen in *Drosophila* cells for cellular factors involved in West Nile virus (WNV) infection (Yasunaga et al., 2014). Among the 50 validated genes found to inhibit WNV infection, dPontin was one of seven genes that were antiviral against additional vector-borne human viruses. By screening additional genes known to be involved in complexes with dPontin, they found that additional components of the Tip60 complex including dReptin, dTip60 and dp400 were antiviral in insects. Further study in mammalian systems revealed that the antiviral function of this complex was conserved, as siRNA-mediated depletion of these genes increased viral infection by multiple human viruses in human cultured cells. While it remains unclear how this complex is antiviral, they showed that interferon-stimulated gene expression induced by polyIC or Sendai virus was intact in these Tip60 complex-depleted cells suggesting a distinct mechanism.

Two studies converge on the role of Pontin and Reptin in cilia biology. Zhaoxia Sun's group (Yale, U.S.A.) investigates the function of cilia in the zebrafish model. From a previous genetic screen for cystic kidney mutants (Amsterdam et al., 2004), they isolated *hi2394*, an insertional mutant displaying kidney cyst formation and ventral body curvature, phenotypes typically associated with ciliary defects in zebrafish. They show that *hi2394* is a loss of function allele of *reptin*. They further provided evidence that *reptin* genetically interacts with known ciliary genes and it is essential for the normal function of cilia (Zhao et al., 2013). In addition, Bernhard Schermer (Cologne, Germany) is working on the pathogenesis of nephronophthisis (NPH), a genetically heterogeneous cystic kidney disease that is the most frequent genetic cause for end-stage renal disease in children and young adults. Several genes have been identified to cause this progressive disorder (NPHP1-17). The gene products, termed the nephrocystins, form protein complexes predominantly at the base of primary cilia. They performed interactome analyses and identified Pontin and Reptin as interactors of NPHP1 and of other ciliopathy proteins. To determine the functional relevance of Pontin *in vivo*, they generated a new mouse model of specific Pontin deletion in kidney tubular epithelial cells that led to severe cystic kidney disease with high perinatal mortality. These studies suggested that Pontin and Reptin are potential novel players in the molecular pathogenesis of cystic kidney disease and related ciliopathies and may provide new mechanistic insights into the pathophysiology of this important group of diseases.

Cancer was another hot topic in the meeting. Owen William's lab (London, UK) has recently shown that Reptin expression was positively regulated by MLL-fusion proteins in acute myeloid leukemia (AML), and that Reptin was a potential target in this disease (Osaki et al., 2013). They are now investigating the pathways regulated by Reptin in AML cells. In this context, Elena Armenteros-Monterroso showed new data suggesting that Reptin regulates c-myc stability in AML cells, further reinforcing the usefulness of using Reptin as a surrogate myc target for therapy.

The group of Jean Rosenbaum (Bordeaux, France) is investigating the functions of Pontin and Reptin in hepatocellular carcinoma where these proteins are overexpressed (Rousseau et al., 2007; Haurie et al., 2009; Menard et al., 2010; Grigoletto et al., 2013). Anne-Aurélie Raymond from this group showed that Reptin silencing in HCC cells reduces the response to DNA double-strand breaks in terms of phosphorylation of H2AX, and BRCA1 and 53BP1 recruitment to the chromatin. Her data suggested that this was secondary to a decreased stability of the H2AX kinase, DNA-PKcs, upon Reptin silencing. Reptin silencing and DNA damage synergized to reduce cell viability. Thus, Reptin is an important cofactor for the repair of DSBs. Overexpression of Reptin in HCC could be a factor of resistance to treatment, consistent with the observed overexpression of Reptin in subgroups of chemo-resistant cancers.

Tommaso Mello (Florence, Italy), in collaboration with Oksana Bereschenko (Perugia, Italy) (Bereshchenko et al., 2012), investigated the onset and progression of hepatocellular carcinoma using a hepatocyte-conditional *Pontin*^+/−^ mouse model and the Diethylnitrosamine cancer induction protocol. Haploinsufficiency of Pontin expectedly resulted in a significant reduction of tumor formation 6 months after DEN injection, compared to *Pontin*^+/+^ mice. Surprisingly, tumor size was significantly larger in *Pontin*^+/−^ mice after 9 and 12 months of cancer progression, showing that although the onset of HCC is delayed, its progression is accelerated. Whereas the underlying molecular mechanisms are still under investigation, this report nevertheless highlights the potential risks of prolonged Pontin inhibition in an intact mammalian organism.

Finally, the interest of pharmaceutical companies in targeting Pontin and Reptin for cancer therapy was clearly shown in the presentation by Takehiko Takata (Daiichi-Sankyo Co. LTD, Japan). Using high-throughput screening, they discovered hit compounds that selectively inhibited the ATPase activity of the Pontin/Reptin protein complex. They then obtained a series of compounds that showed *in vitro* anti-proliferative activities against human cancer cell lines that correlated well with Pontin/Reptin ATPase inhibitory activities. Oral administration of the compounds exhibited potent *in vivo* antitumor effects on human tumors subcutaneously xenografted into immuno-deficient mice, without severe toxicity.

## Summary

This very successful 2nd meeting has highlighted the impressive advance in our knowledge of Pontin and Reptin. Although many outstanding questions and controversies remain, we are confident that they will be addressed in the coming years by a growing number of research groups interested in these proteins. These questions revolve around pinpointing the exact function or functions of these proteins in the cell. To date, it remains unclear how they can exert such a global influence on the cell physiology and how they interact with a multitude of proteins to carry out these functions. Determining the molecular structures of Pontin/Reptin in isolation and as part of several complexes in the presence of different nucleotides will allow a clearer understanding of the molecular basis of their activity. Furthermore, many groups and pharmaceutical companies are currently addressing the therapeutic value of inhibiting these proteins as a potential for developing novel anticancer drugs, and we expect this potential will be fully realized in the near future. Therefore, there is much to look forward to for the 3rd International Workshop that will be organized in Madrid in 2016 by Oscar Llorca and his team.

### Conflict of interest statement

The authors declare that the research was conducted in the absence of any commercial or financial relationships that could be construed as a potential conflict of interest.
